# Volume-based structural connectome of epilepsy partialis continua in Rasmussen’s encephalitis

**DOI:** 10.1093/braincomms/fcae316

**Published:** 2024-09-20

**Authors:** Cong Fu, Xue Yang, Mengyang Wang, Xiongfei Wang, Chongyang Tang, Guoming Luan

**Affiliations:** Department of Neurosurgery, Epilepsy Center, Sanbo Brain Hospital, Capital Medical University, Beijing 100093, China; Basic—Clinical Joint laboratory, Capital Medical University, Beijing 100093, China; Department of Neurosurgery, Epilepsy Center, Sanbo Brain Hospital, Capital Medical University, Beijing 100093, China; Basic—Clinical Joint laboratory, Capital Medical University, Beijing 100093, China; Department of Neurology, Epilepsy Center, Sanbo Brain Hospital, Capital Medical University, Beijing 100093, China; Epilepsy Institution, Beijing Institute of Brain Disorders, Beijing 100069, China; Department of Neurosurgery, Epilepsy Center, Sanbo Brain Hospital, Capital Medical University, Beijing 100093, China; Basic—Clinical Joint laboratory, Capital Medical University, Beijing 100093, China; Department of Neurosurgery, Epilepsy Center, Sanbo Brain Hospital, Capital Medical University, Beijing 100093, China; Basic—Clinical Joint laboratory, Capital Medical University, Beijing 100093, China; Department of Neurosurgery, Epilepsy Center, Sanbo Brain Hospital, Capital Medical University, Beijing 100093, China; Basic—Clinical Joint laboratory, Capital Medical University, Beijing 100093, China; Epilepsy Institution, Beijing Institute of Brain Disorders, Beijing 100069, China

**Keywords:** Rasmussen’s encephalitis, rare epilepsy, epilepsy partialis continua, hemispheric atrophy, structural connectome

## Abstract

Rasmussen's encephalitis is a rare, progressive neurological inflammatory with hemispheric brain atrophy. Epilepsy partialis continua (EPC) is a diagnostic clinical condition in patients with Rasmussen's encephalitis. However, the incidence of EPC in the natural course of Rasmussen's encephalitis is only about 50%. The majority of experts hold the belief that EPC is associated with dysfunction in the motor cortex, yet the whole pathogenesis remains unclear. We hypothesize that there is a characteristic topological discrepancy between groups with EPC and without EPC from the perspective of structural connectome. To this end, we described the structural MRI findings of 20 Rasmussen's encephalitis cases, 11 of which had EPC, and 9 of which did not have EPC (NEPC), and 20 healthy controls. We performed voxel-based morphometry to evaluate the alterations of grey matter volume. Using a volume-based structural covariant network, the hub distribution and modularity were studied at the group level. Based on the radiomic features, an individual radiomics structural similarity network was constructed for global topological properties, such as small-world index, higher path length, and clustering coefficient. And then, the Pearson correlation was used to delineate the association between duration and topology properties. In the both EPC and NEPC groups, the volume of the motor cortex on the affected side was significantly decreased, but putamen atrophy was most pronounced in the EPC group. Hubs in the EPC group consisted of the executive network, and the contralateral putamen was the hub in the NEPC group with the highest betweenness centrality. Compared to the NEPC, the EPC showed a higher path length and clustering coefficient in the structural similarity network. Moreover, the function of morphological network integration in EPC patients was diminished as the duration of Rasmussen's encephalitis increased. Our study indicates that motor cortex atrophy may not be directly related to EPC patients. Whereas atrophy of the putamen, and a more regularized configuration may contribute to the generation of EPC. The findings further suggest that the putamen could potentially serve as a viable target for controlling EPC in patients with Rasmussen's encephalitis.

## Teaser

Fu *et al*. report that putamen atrophy was most pronounced in Rasmussen's Encephalitis patients with epilepsia partialis continua and a more regularized network configuration may contribute to the generation of epilepsia partialis continua.

## Graphical Abstract

**Graphical Abstract:**
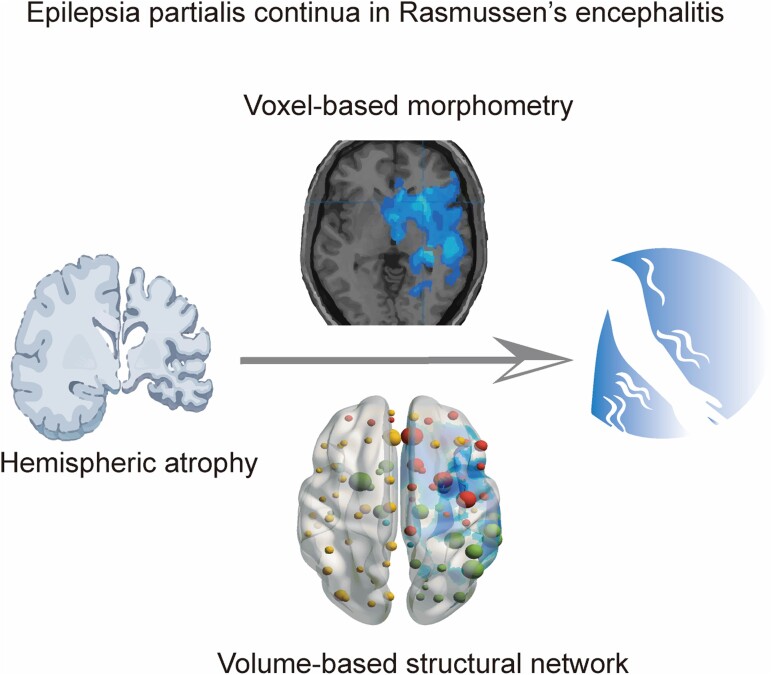

## Introduction

Rasmussen’s encephalitis (RE) is a rare immune-mediated disease, presenting with epilepsy partialis continua (EPC) and progressive unilateral cerebral atrophy with concurrent neurologic dysfunction. The onset is marked, in almost all cases, by focal or focal-to-bilateral tonic–clonic seizures. Simple focal motor (SFM) seizures are the most common (77% of cases), followed by focal-to-bilateral tonic–clonic seizures and complex focal seizures.^1^ Motor epilepsy partialis continua, an important criterion for RE diagnosis, is a widely described variant of SFM status epilepticus. Recurrent myoclonic convulsions are typical clinical symptoms of EPC and are thought to cause hemiplegia or serious cortical defects. However, it is interesting to note that nearly 50% of children are without EPC even in the acute phase of RE.^2^ At present, all authors agree that the contribution of primary motor areas is indispensable for EPC generation.^3^ And MRI and electrophysiological studies suggested that RE patients also showed cortical dysfunction with other cortical areas.^4-6^ Thus, the motor-related cortical network of RE patients with EPC symptoms may be different from that of other patients who suffer from less frequent SFM seizures.

The advent of the epileptic network has shifted the focus to the affected entire neural networks, rather than an isolated focal brain pathology, to deeply understand the seizure mechanism.^7^ Based on the morphology of cerebral grey matter, the structural connectome can be inferred by studying the covariance of morphology using graph theory.^8^ Such graph-based structural network studies capture an important aspect that is crucial for understanding the pathophysiology of epileptogenesis.^9^ To date, many RE morphometry studies have used structural magnetic resonance imaging (MRI) to investigate alterations in regional grey matter volume (GMV) due to predominant GM atrophy and hyperintense signals in the acute phase of RE.^10^ Using voxel-based morphometry (VBM) analysis, extensive atrophy of the affected hemisphere has been confirmed,^5,11^ and regional atrophy also occurs in the contralateral hemisphere of RE.^12^ However, few studies have addressed the discrepancy of high-frequency seizures in the acute phase of RE, which may help us study the role of motor cortex and cortical disorganization in RE patients with EPC or without EPC (NEPC). Thus, we hypothesized that EPC would show a disorganized structural connectome that is not restricted to motor cortex.

To test this hypothesis, we assessed GMV-based network property differences between EPC and NEPC groups by constructing a traditional structural covariant network (SCN) from a group-level perspective. Considering the rarity of RE patients and individual heterogeneity, an individual radiomics-based structural similarity network (iRSSN) was constructed for each RE patient.^13^

## Materials and methods

### Subjects

**Figure 1:**
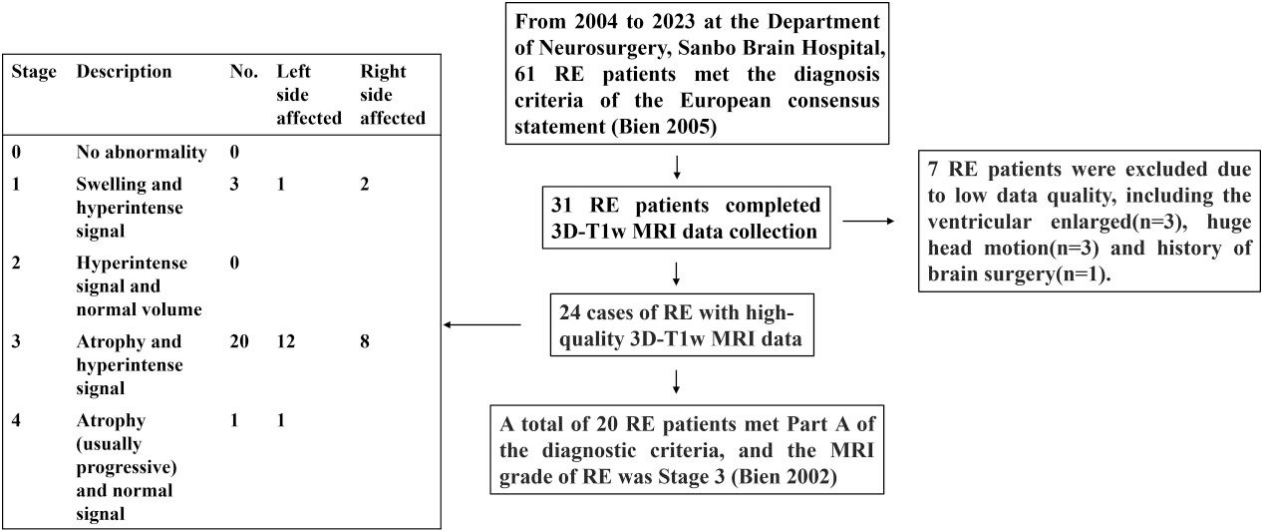
**Patients enrolled workflow.** Sixty-one patients with RE were diagnosed and treated in our centre from September 2004 to May 2023. After the screening of data quality and MRI grade of RE, 20 RE patients were included for further study. RE, Rasmussen encephalitis.

We conducted a retrospective case-note review of 61 RE patients who underwent presurgical multidisciplinary evaluation at our centre from September 2004 to May 2023 at the Department of Neurosurgery, Sanbo Brain Hospital, Capital Medical University. The inclusion criteria were as follows: (i) RE patients diagnosed according to the diagnostic criteria Part A of Bien *et al*.^14^ (Supplementary Table 1); (ii) the median age of RE onset is 6 years^15,16^ and there were no patients with adult-onset RE in our center, thus we only included childhood-onset RE patients. Through multistep screening and selection in Fig. 1, 20 RE patients with high-quality magnetic resonance imaging (MRI) data were included for further study. According to the following definition of EPC,^17^ ‘EPC is defined as spontaneous regular or irregular clonic muscular twitching affecting a limited part of the body, sometimes aggravated by action or sensory stimuli, occurring for a minimum of one hour, and recurring at intervals of no more than ten seconds’, RE patients were divided into the EPC group (*N* = 11) and the NEPC group (*N* = 9). The clinical characteristics of patients in the RE group are shown in Supplementary Table 2.

Twenty healthy subjects who were brain MRI-negative were recruited and matched for sex and age. And the details of their demographic information are shown in Supplementary Table 3. All participants or their guardians signed the informed consent form.

### MRI data acquisition

Magnetic resonance imaging data were obtained for all participants. Briefly, MR images were acquired using standard 3.0 T MRI scanners GE and 1.5T MRI scanner Philips. Overall, 20 scans (4 EPC scans, 4 NEPC scans, and 10 control scans) were performed with a 1.5 T Philips Achieva scanner. The other 20 scans were performed with a 3.0 T GE DISCOVERY MR750W scanner. The scanner effect is included as a covariate throughout the analysis. Whole-brain T1-weighted image datasets were acquired using Magnetization Prepared Rapid Acquisition with Gradient Echo (MPRAGE) sequence in 1.5 T scanner (echo time (TE) = 9.2 ms, repetition time(TR) = 25 ms, flip angle (FA) = 30 degrees, matrix = 256 × 256, field of view (FOV) = 100, slice thickness = 0.94 mm, slices = 213), as well as in 3.0 T scanner (TE = 3.2 ms, TR = 8.7 ms, and FA = 12 degrees, matrix = 256 × 256, FOV = 100, slice thickness = 1 mm, and slices = 176).

### Voxel-based morphometry in grey matter volume

**Figure 2:**
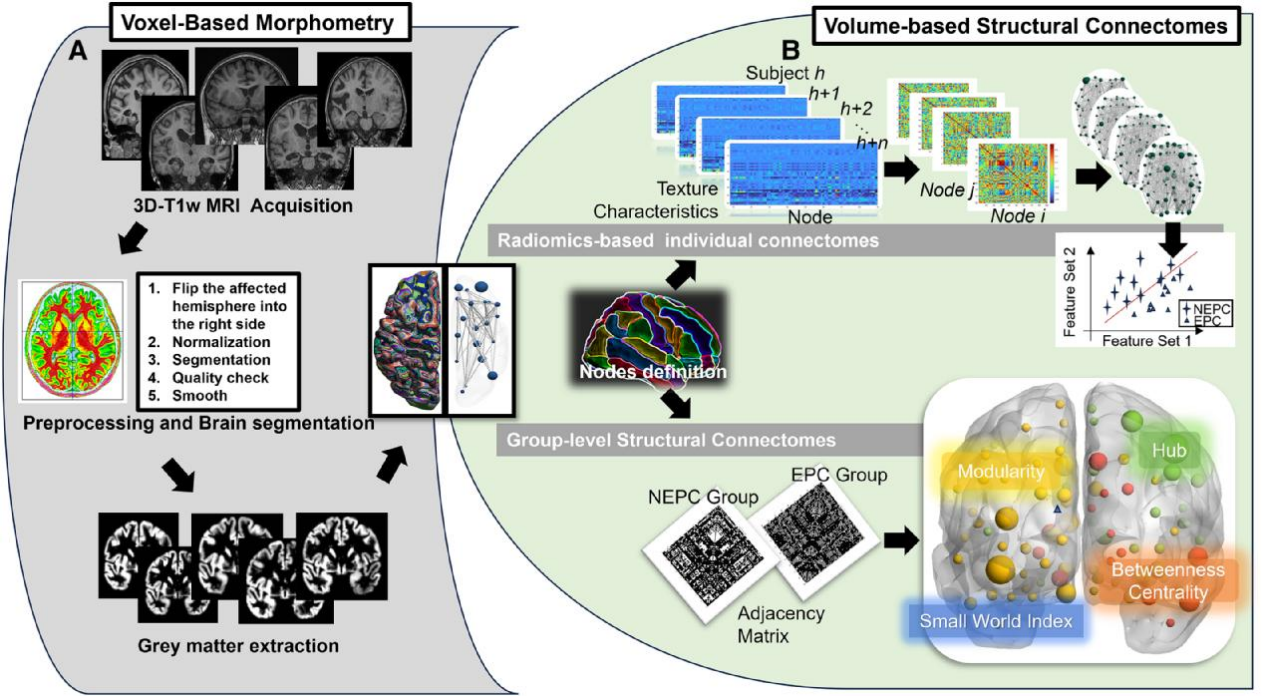
**Steps in processing the volume-based connectome.** (**A**) Grey matter reconstruction was carried out using a software program. (**B**) The AAL atlas was used for parcellating the grey matter to 90 regions (45 on each hemisphere). The computation of radiomics features in each brain region and feature matrix of radiomics features. Network construction with Pearson correlation. The calculation of network properties and their correlation with clinical variates. Binary adjacency matrices were conducted between regional gyrification across individuals within EPC, NEPC, and control study groups separately. The network parameters were calculated including hubs and modularity. EPC, epilepsy partialis continua; NEPC, patients without EPC; AAL, Automated Anatomical Labelling.

All images were checked and flipped as needed to ensure that the affected hemisphere was located on the same side (the right hemisphere) using SPM12 (https://www.fil.ion.ucl.ac.uk/spm/software/spm12/). The Computational Anatomy Toolbox (CAT12) (http://www.neuro.uni-jena.de/cat/), which runs within SPM12, was used for cortical and subcortical volume segmentation from T1-weighted images in a fully automated fashion. First, images were transformed into standard space using a 12-parameter affine-only linear transformation and segmented into three tissue classes representing grey matter, white matter, and cerebrospinal fluid, followed by visual inspection for quality control at various processing steps. The resulting segmented maps were then smoothed using an 8-mm full-width at half-maximum Gaussian kernel (Fig. 2A).

Voxel-based morphometry statistics were conducted in SPM12 using one-way analysis of variance (ANOVA) with the main effect of diagnosis (EPC, NEPC, and control groups) while using age, sex, and total intracranial volume (TIV) as covariates to remove their effects on variance. We assessed the main effect of diagnosis using *F* contrasts. Differences meeting a voxel-level threshold of *P* < 0.001 (without correction) and a cluster size criterion > 100 voxels were deemed significant. In the DPABI toolbox (http://rfmri.org/DPABI), Gaussian random field (GRF) theory was applied for *post hoc* multiple comparison corrections.^18^ Statistical maps were generated, and a strict threshold was applied (voxelwise *P* < 0.001 and clustewise *P* < 0.05, two-tailed).

### Structural network construction

Parcellated 90 brain regions and labels abbreviation was based on Automated Anatomical Labelling (AAL) Atlas (Supplementary Table 4). For group-level SCN construction, a 90 × 90 correlation matrix (Pearson correlation) was generated based on the GMV results for each of the three groups (Fig. 2B). For the iRSSN, 43 representative high-order Radiomics features were selected to obtain the multiview structural information,^13,19,20^ including Grey-level co-occurrence matrix (GLCM), Grey-level run-length matrix (GLRLM), Grey-level size zone matrix (GLSZM), and Neighbourhood grey-tone difference matrix (NGTDM). And then Pearson correlation coefficients were calculated between each pairwise ROIs, providing an individual wise 90 × 90 distance matrix for the 3 study groups (Fig. 2B). The whole steps of iRSSN construction were the same as those of Liu *et al*.,^21^ and the open-source code was also provided (https://github.com/zhema620/iRSSN; see Supplemental Methods for more information). A Graph Analysis Toolbox^22^ was used for studying various topological properties.^22,23^ For the weighted connectivity matrices obtained from each individual, a range of network thresholds based on connection density (i.e. 0.20–0.46, with interval steps of 0.02) was applied to generate binary undirected adjacency matrices. This choice of range enabled between-group comparisons in topological measures across graphs with a comparable number of edges but without inducing disconnection or losing small-worldness. Topological measures were normalized to equivalent values derived from 20 random (‘null’) networks with the same degree of distribution.

### Statistical analysis

Global integration, regional segregation, and small world (σ) were measured, including global efficiency (Eg), normalized/global clustering coefficient (γ/Cp), normalized/global characteristic path length (λ/Lp), betweenness centrality (Bc), hubs, and modularity. Measures were defined as in previous studies^24^ and were quantified using GAT with SPM12 running on MATLAB2022b. See the Supplemental Methods for formal descriptions of these properties.

The network measures were calculated for each network at each density and summarized using the area under the curve (AUC).^25^ The Anderson–Darling test was used for the normality test. A 1-way ANOVA was used in the iRSSN comparison, followed by *post hoc t*-tests to compare the AUC among the three study groups. Bonferroni-corrected two-tailed AUC*p* = 0.05 was chosen as the threshold of statistical significance and subsequently converted into Cohen’s *d* effect sizes for interpretation. The VBM and GAT analyses were repeated more than twice with the same results. BrainNet viewer^26^ and Cytoscape (https://cytoscape.org/) were used for network visualization.

The two-tailed Pearson correlation coefficient test was used to assess the association between topological measures and clinical variables (age at diagnosis, age at onset, and disease duration). The significance level was set at *P* < 0.05. These correlations were tested for each patient group separately.

## Results

### Demographic and clinical features

As shown in Table 1, the mean ages of the RE (EPC and NEPC) and control groups were 7.4 (SD, 4.2), 8.2 (SD, 4.1), and 10.4 (SD, 4.1) years, respectively. The sexes (N, female) of the RE (EPC and NEPC) and control groups were 11 (8), 9 (6), and 20 (10), respectively. There were no significant differences in the mean ages, sex, or intracranial volume among the three groups. In addition, the results revealed no significant differences in age at onset, disease duration, hemisphere involved, hippocamps sclerosis, or number of AED between the two groups of RE patients.

**Table 1:** Participant characteristics

Variable	RE(*n* = 20)		HC(*n* = 20)	Group Statistic	*P*
	EPC(*n* = 11)	NEPC(*n* = 9)			
Age, mean (SD)	7.364 (4.225)	8.222(4.086)	10.350(4.107)	*F* *=* 2.083	0.139
Sex (No. females)	8	6	10	*χ2* = 1.742	0.418
Intracranial volume, mean (SD)	1337(144.000)	1353(94.060)	1422(150.4)	*F* = 1.614	0.213
Age at onset (years), mean (SD)	4.909(2.071)	5.000(1.414)	NA	*t* = 0.112	0.912
Disease duration (months), mean (SD)	26.82(34.590)	36.67(43.630)	NA	*t* = 0.564	0.580
Hemisphere involved (No. right)	4	5	NA		0.653
Hippocamps sclerosis (No.)	3	5	NA		0.362
AEDs, mean (SD)	3.091(1.300)	2.444(1.014)	NA	*t* = 1.217	0.239

Age of EPC and NEPC groups was the age at diagnosis. The differences among the three groups were evaluated using Pearson’s χ2 test for sex, ANOVA for intracranial volume, two-sample *t-test* for RE clinical continue variables, and Fisher’s exact test for categorical variables with SPSS 25.0. SD, standard deviation; AEDs, antiepileptic drugs; No., number; NA, not applicable.

### Group differences in GMV

**Figure 3:**
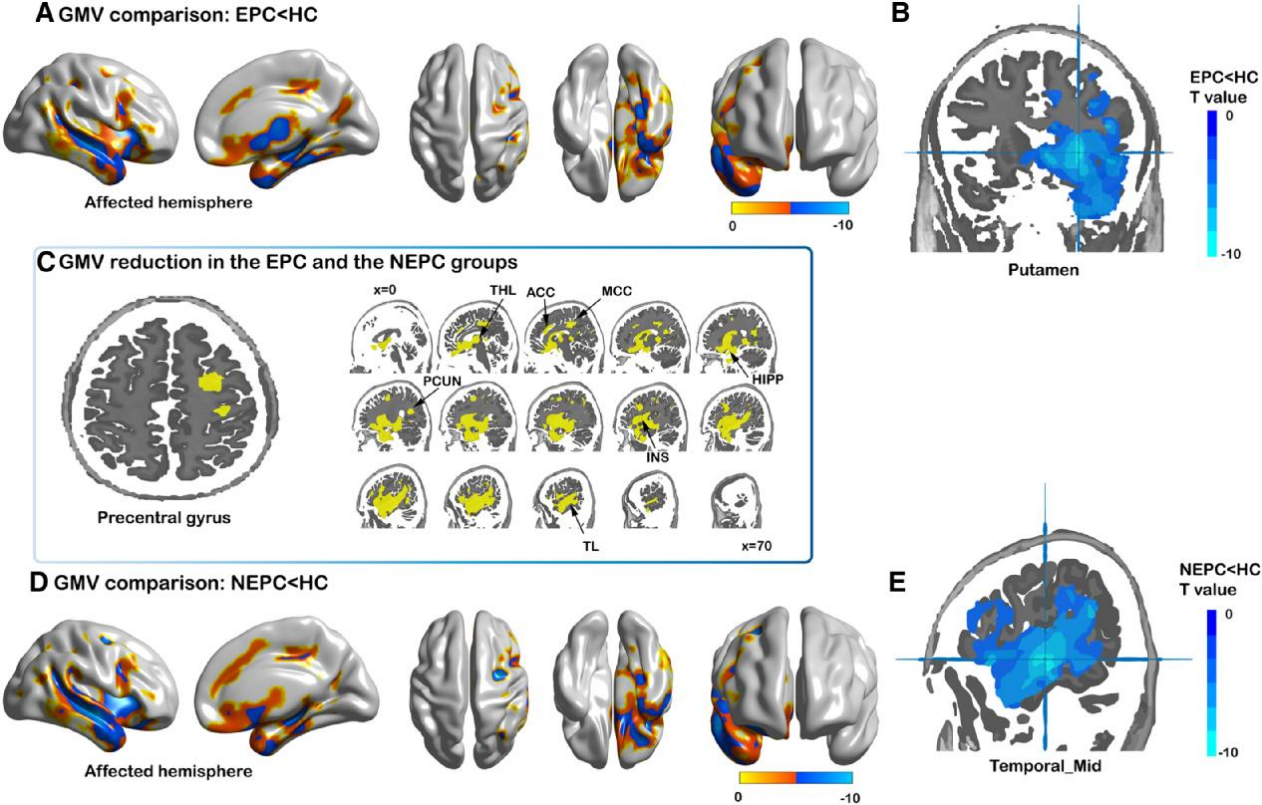
**GMV results.** (**A-B**) The EPC group (*N* = 11) showed a wide volume reduction compared to the control group, mainly around the perisylvian region and the putamen (*t* = −9.18, *P* < 0.001) showed the largest volume difference. (**C**) Compared with controls, precentral gyrus, temporal lobe, PCUN, MCC, and ACC on the affected side presented a common reduction in both RE groups. (**D-E**) In the NEPC group (*N* = 9), the largest cluster was the Temporal_Mid (*t* = −9.88, *P* < 0.001) with the highest difference. The colour bar represents the *t* values of the group analysis. ACC, cingulum_ant cortex; MCC, cingulum_mid cortex; PCUN, precuneus; TL, temporal lobe; INS, insular; HIPP, hippocampus; THL, thalamus.

Compared with the control group, there was no significant increase in GMV or marked decrease on the unaffected side in either RE group. And both EPC group (*N* = 11) and NEPC (*N* = 9) group showed a significant decrease in the precentral gyrus on the affected side (*t* = −6.99, *P_corrected_* < 0.01; *t* = 6.32, *P_corrected_* < 0.01). The EPC group showed a wide volume reduction in the affected side included in the temporal lobe, insular, and subcortical GM, among which the putamen showed the largest volume difference (*t* = −9.18, *P_corrected_* < 0.01). We also found significant decreases in the middle cingulum (*t* = −6.14, *P_corrected_* < 0.01), precuneus (*t* = −5.96, *P_corrected_* < 0.01), anterior cingulum (*t* = −4.71, *P_corrected_* < 0.01), and middle frontal gyrus [*t* = −4.50, *P_corrected_* < 0.01 (see Fig. 3A)]. In the NEPC results, the largest cluster was also in the perisylvian region and the middle of the temporal gyrus with the highest difference (*t* = −9.88, *P_corrected_* < 0.01). And the largest cluster had other local maxima in the putamen (*t* = −7.12, *P_corrected_* < 0.01). In addition, the middle frontal gyrus (*t* = 9.04, *P_corrected_* < 0.01), superior frontal gyrus (*t* = −5.14, *P_corrected_* < 0.01), and middle cingulum (*t* = −6.25, *P_corrected_* < 0.01) had significant loss of volume on the affected side in the NEPC group (see Fig. 3B). However, there were no differences in GM volumes in patients with EPC compared to patients without EPC, neither at a conservative threshold of *P* < 0.05 Gaussian random-field (GRF) corrected nor at a more liberal threshold of *P* < 0.001 uncorrected. Raw volume results for the 90 GM brain regions among the three groups are shown in Supplementary Table 5. The details of the group differences are shown in Supplementary Table 6.

### Network properties in SCN

**Figure 4:**
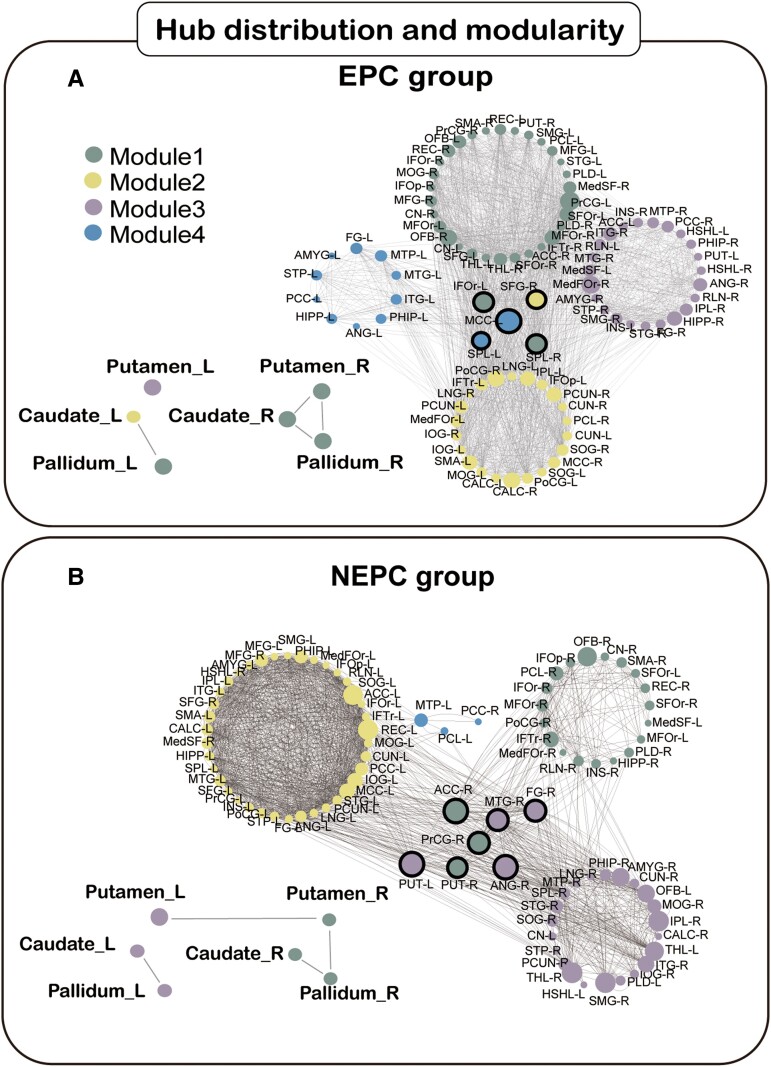
**GMV connectome results.** Topological representation (minimizing free energy) for EPC (**A**) and NEPC (**B**) population networks. And the hub regions were shown in the middle of the topological network represented by the black circle. Network connection of Subcortical nuclei was displayed in the undirected graphs. Brain regions' abbreviation was shown in Supplementary Table 4.

We investigated between-group differences in SCN measures, comparing the AUC for these network measure curves (density range of 0.20:0.02:0.46). Our qualitative analysis of the hub distribution using nodal Bc showed that cortical regions were classified into primary, association, and paralimbic areas.^27^ In the EPC group, 3/5 hubs were observed in association, and the rest of the hubs were in paralimbic areas. Moreover, all hubs were composed of nodes with bilateral central executive networks (CEN)^28^ in Fig. 4A. In the NEPC group, the 6/7 hubs were lateralized on the affected side except the contralateral putamen (Fig. 4B and Supplementary Table 7).

In addition, there were marked differences in the composition and topological roles of modules. The network for the EPC group comprised 4 connected modules, which varied in size from 12 to 32 regional nodes (see Fig. 4A). The largest module with 32 nodes presented multilobe participation and mainly frontal patterns on the affected side. The second largest module (23 regions) was a parieto-occipital pattern that mainly included bilateral postcentral, precuneus, and occipital lobe structures. The other 23-node module was a tempo-limbic-paralimbic pattern consisting of the temporal lobes, hippocampus, parahippocampus, and amygdala, which was limited to the affected hemisphere. Symmetrically, a tempo-mesial module (12 regions) was observed on the unaffected side.

The NEPC network also comprised four connected modules, 23, 38, 26, and 3 nodes in each module (see Fig. 4B). First, the affected hemisphere was divided into two modules by the postcentral sulcus, 23 nodes in the frontoparietal module and 26 nodes in the temporo-occipital module. Importantly, we found that bilateral putamen had structural connection in the minimal network density while the putamen connection was not observed in the EPC group. The regional nodes of the largest module (38 nodes) were all on the unaffected hemisphere, and the minimal module (three nodes) consisted of the post cingulum, paracentral lobule, and temporal pole on the unaffected hemisphere. Details results are shown in Supplementary Table 8.

### Network properties in iRSSN

**Figure 5:**
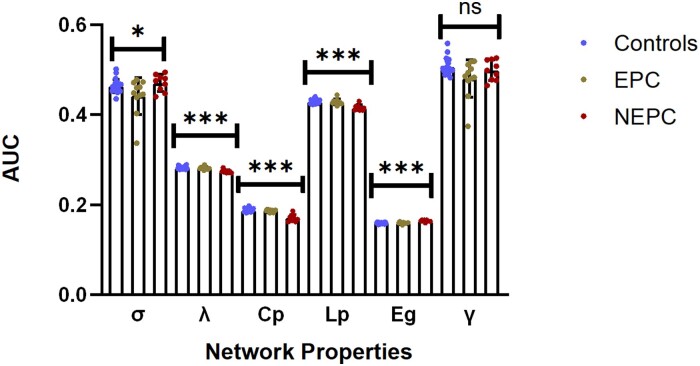
**Network properties comparisons for the HC, EPC, and NEPC groups.** The AUC mean scores of each graph variable were estimated with the selected density range of 0.2–0.46 in the HC (*N* = 20), EPC (*N* = 11), and NEPC (*N* = 9) groups. The bar graphs show the differences among these groups. Statistical significance was analysed by using the one-way ANOVA and the *post hoc* test (n.s. = not significant; *P < 0.05, ***P < 0.0001). There were five graph properties showing significant differences among three groups: σ(*F_(2,37)_* = 3.95), λ (*F_(2,37)_* = 17.70), Cp (*F_(2,37)_* = 36.43), Lp (*F_(2,37)_* = 18.94), and Eg (*F_(2,37)_* = 21.15). Data are presented as mean ± SD. σ, small-world index; Eg, global efficiency; γ/Cp, normalized/global clustering coefficient; λ/Lp, normalized/global characteristic path length.

We noted statistically significant group differences (*P* < 0.05) in the global topological properties of σ(AUC*_F(2,37)_* = 3.95, AUC*_P_* < 0.05), λ (AUC*_F(2,37)_* = 17.70, AUC*_P_* < 0.0001), Cp (AUC*_F(2,37)_* = 36.43, AUC*_P_* < 0.0001), Lp (AUC*_F(2,37)_* = 18.94, AUC*_P_* <0.0001), and Eg (AUC*_F(2,37)_* = 21.15, AUC*_P_* < 0.0001). *Post hoc* comparisons revealed that graph properties were significantly different when the HC(*N* = 20) or EPC(*N* = 11) group was compared with the NEPC (*N* = 9) group. The details were as follows: σ(NEPC versus EPC: *t* = 2.55, *P* = 0.45); λ (HC versus NEPC: *t* = 5.78, *P* < 0.0001; NEPC versus EPC: *t* = 4.67, *P* = 0.001), Lp (HC versus NEPC: *t* = 5.92, *P* < 0.0001; NEPC versus EPC: *t* = 4.98, *P* < 0.0001) and Cp (HC versus NEPC: *t* = 8.29, *P* < 0.0001; NEPC versus EPC: *t* = 6.70, *P* < 0.0001), and Eg (HC versus NEPC: *t* = 8.85, *P* < 0.0001; NEPC versus EPC: *t* = 7.42, *P* < 0.0001). Importantly, there were no significant differences in the EPC group compared with the HC group. The AUC results of the global network properties within the selected density range (0.20:0.02:0.46) are shown in Fig. 5 and Table 2.

**Table 2:** Network properties in the HC, EPC, and NEPC groups

Network Properties	AUC Mean (SD)			AUC*_F_* score	AUC*_P_* value	HC versus EPC		HC versus NEPC		EPC versus NEPC	
	HC (*n* = 20)	EPC (*n* = 11)	NEPC (*n* = 9)			AUC*_P_*	Cohen’s *d*	AUC*_P_*	Cohen’s *d*	AUC*_P_*	Cohen’s *d*
γ	0.506 (0.019)	0.480 (0.041)	0.498 (0.023)	3.228	-	*	0.523	-	1.403	-	0.191
λ	0.282 (0.003)	0.281 (0.003)	0.274 (0.004)	17.70	****	-	−0.045	****	2.131	****	1.423
σ	0.463 (0.015)	0.442 (0.041)	0.470 (0.019)	3.951	-	-	0.387	-	0.109	*	−0.256
Cp	0.188 (0.005)	0.187 (0.003)	0.171 (0.008)	36.43	****	-	0.274	****	3.361	****	2.795
Lp	0.429 (0.005)	0.428 (0.006)	0.416 (0.006)	18.94	****	-	−0.111	****	2.363	****	1.484
Eg	0.160 (0.002)	0.160 (0.002)	0.164 (0.003)	21.15	****	-	0.071	****	−2.434	****	−1.624

The ‘-’ symbol indicates that there is no statistically significant differences in network properties. **** AUC*_P_* < 0.0001, * AUC*_P_* = 0.01∼0.05. γ , normalized clustering coefficient; λ, normalized path length; σ, small-world index; Cp, clustering coefficient; Lp, characteristic path length; Eg, global efficient.

### Association between network properties and clinical information

**Figure 6:**
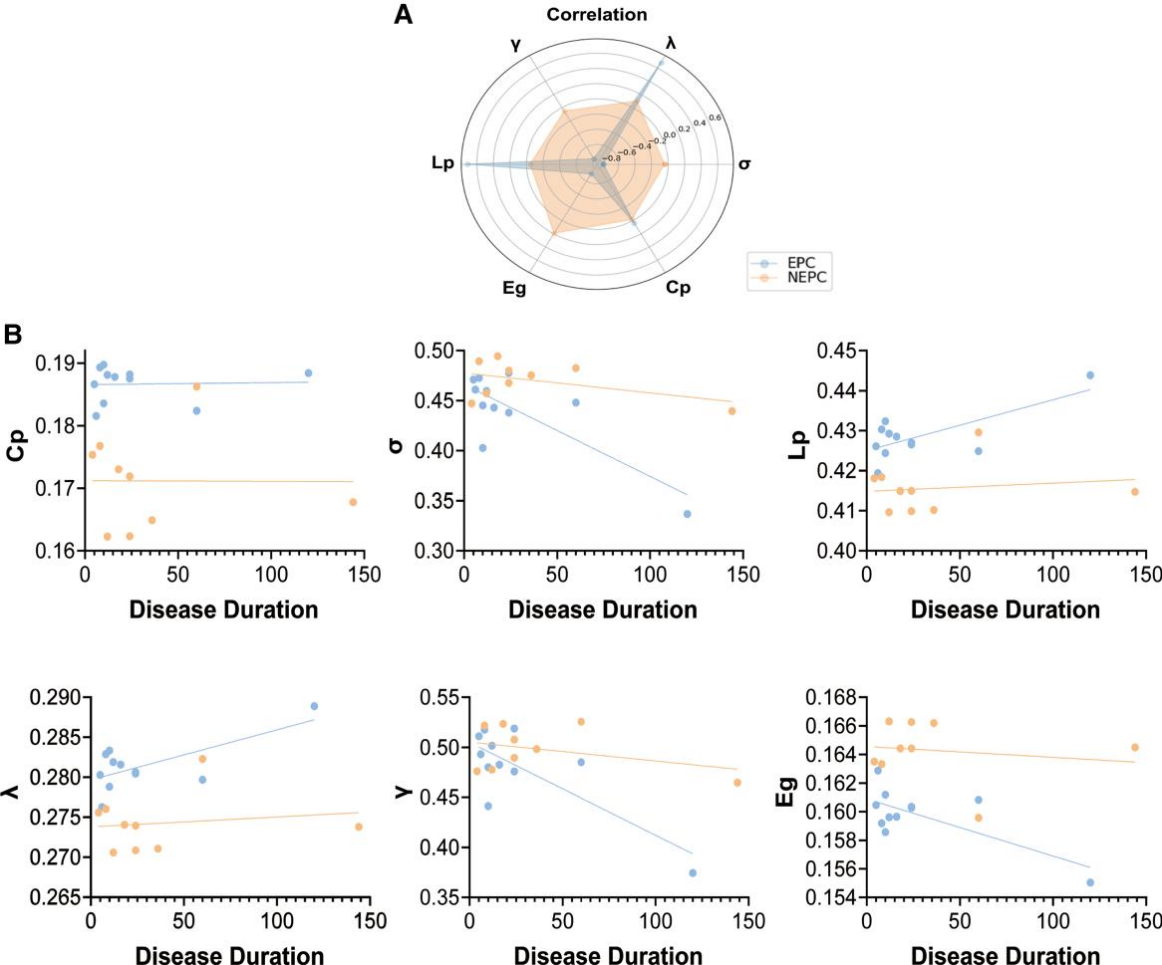
**Association between graph metrics and RE duration.** (**A**) A radar graph showed the association between the disease duration and six topological properties in the EPC group (*N* = 11) and the NEPC group (*N* = 9). And the value of Pearson’s correlation coefficient *R* was displayed in the radar graph. (**B**) With the exception of Cp in the EPC group, there was a strong correlation between RE duration and five topological properties: σ (*R* = −0.783, *P* = 0.004), λ (*R* = 0.683, *P* = 0.021), γ (*R* = −0.775, *P* = 0.005), Lp (*R* = 0.714, *P* = 0.014), Eg (*R* = −0.708, *P* = 0.015). However, there was no significant correlation between disease duration and network metrics in the NEPC group. σ, small-world index; Eg, global efficiency; γ/Cp, normalized/global clustering coefficient; λ/Lp, normalized/global characteristic path length.

Associations between clinical information (age of onset, disease duration, and age at diagnosis) and the six network measures were tested for each patient group (EPC and NEPC) separately (Supplementary Table 9). The age of onset was not significantly associated with graphic properties in the EPC(*N* = 11) and NEPC(*N* = 9) groups. In the EPC group (see Fig. 6), σ showed a significant negative correlation with disease duration (*r* = −0.783, *P* = 0.004) and age at diagnosis (*r* = −0.674, *P* = 0.023). In addition, the global segregation metric γ was also negatively correlated with disease duration (*r* = −0.775, *P* = 0.005) and age at diagnosis (*r* = −0.674, *P* = 0.023). For the ability of network integration, λ and Lp showed a significant positive correlation with disease duration (*r* = 0.683, *P* = 0.021 and *r* = 0.714, *P* = 0.014 respectively). With a longer duration of RE disease, the EPC patients showed a lower global efficiency Eg (*r* = −0.708, *P* = 0.015). However, there were no significant correlations between clinical variables and network metrics in the NEPC group.

## Discussion

To our knowledge, this is the first graph-based volumetric connectome study to distinguish the disorganized network pattern of EPC and NEPC in patients with RE. We had 4 major findings. First, we found that loss volume in the motor cortex was present in both RE groups, and putamen atrophy was most pronounced in the EPC group. Moreover, the volume decreases in subcortical nuclei, such as the putamen and caudate nucleus in the EPC group, were greater than those in the NEPC group. Second, the contralesional putamen nucleus was the hub node in the posthead module on the affected side. Moreover, an interhemispheric separation of the temporolimbic system was observed in EPC modularity, while an intrahemispheric division was observed on the affected side of the NEPC group split at the postcentral sulcus. Third, global network analysis of iRSSN revealed higher Cp and Lp in RE patients with EPC, suggesting a shift towards network regularization. Conversely, the NEPC group showed lower Cp and Lp with significant differences in the global network metric, indicating a randomized configuration. Last, the RE duration in the EPC group was associated with γ, λ, σ, Lp, and Eg, whereas that of the NEPC group was not associated with these parameters.

### Voxel-based GMV alterations

Similar to the study of Larionov *et al*.,^6,29^ in our study, the GMV loss of the affected side was significantly higher than that of controls. Referring to cortical atrophy differences, our results show that the precentral gyrus, perisylvian region, and the frontal lobe were preferentially involved in RE, which is entirely compatible with previous studies.^5,6^ Regarding subcortical GMV reduction, we found that the putamen of the affected side showed a significant change in the EPC group. Wagner *et al*. presented the same results that the putamen was one of the most severe atrophies in the subcortical, using the ratio of the volume of the affected side to the unaffected side. Moreover, this finding is supported by a study by Rajesh *et al*., who found predominant atrophy of the putamen nucleus in a group of 12 RE.^30^ However, the above studies did not consider the effect on the frequency of motor seizures. In a diffusion tensor imaging (DTI) study of patients with epilepsy, the results suggest that recurrent seizures may lead to progressive microstructural putamen alterations,^31^ which could explain our results that the volume of the putamen on the affected side showed a greater reduction in the EPC group than the NEPC group. Bouilleret *et al*. found that a reduction in L-DOPA uptake was found in the bilateral putamen and caudate nucleus in temporal lobe epilepsy (TLE) patients.^32^ An epileptic monkey study has provided evidence that the electrophysiological activity of putamen cells increases the duration of motor seizures.^33^ This finding suggests that the anatomical and functional impairment of the putamen may result from recurrent seizures. More importantly, the affected putamen showed maximal atrophy compared to controls in grey matter, implying a putamen-dominated atrophy network in the EPC group.

### Network properties in SCN

In the EPC group, the hub collection was all CEN-related areas distributed in both hemispheres, avoiding motor-related atrophy areas. According to sophisticated electrophysiological studies, the origin of EPC has been linked to the motor cortex.^17^ However, in our EPC group, the primary motor cortex had wide morphological involvement and developed into a state of disorganization and disconnection. Therefore, the SCN may be seen as a network hub. In the functional imaging data, the CEN system with central hubs in the prefrontal and posterior parietal cortex was associated with goal-directed behaviour and has been shown to be involved in higher-order cognitive processes.^34^ The hubs related to CEN may be the results of uncontrolled seizure events in RE patients with EPC.

Compared with the bilateral distributed hubs in the EPC group, hubs of the NEPC group were limited to the affected side, except the putamen, implying that the affected side dominated the topological pattern. The bilateral putamen acted as a network hub with higher Bc, playing an important role in epileptic network communication in the NEPC group. Our results showed that the hyperactivity putamen had a connection with the pallidum on the affected side, suggesting increasing inhibition from the reduced inhibition from the putamen to the pallidus. To our knowledge, the striatum (putamen and caudate nucleus) is not directly involved in the generation of seizures; rather, it plays an important role in the propagation and control of motor seizures.^35^ In the direct pathway, the striatum projects inhibitory GABAergic efferent connections directly to the internus globus pallidus through the striatal-thalamo-cortical circuit, leading to an antileptic effect on motor seizure.^36^ Thus, it was possible to control motor seizures in the NEPC group of patients with RE and indicated that the NEPC group needed more compensation from the putamen rather than the caudate than the EPC group. Network hubs are preferentially targeted due to their topological centrality and high metabolic demand.^37^ This may be the reason why the EPC group showed more volume loss in the affected putamen, implying that the putamen was more vulnerable and compensation priority. Mar Carreño *et al*. reported an adult RE patient with focal dystonia and the good response to unilateral globus pallidus internus DBS.^38^ Thus, it is possible that DBS could be used to treat successfully for RE patients with motor seizures. Taken together, the hub putamen may be a useful DBS target for RE patients with EPC.

### Network properties in iRSSN

We performed connectome analysis on the MRI-based GMV of RE patients with EPC, RE patients with NEPC, and healthy controls at the group level. However, in recent years, many studies have focused on the individual-level morphological similarity network.^39,40^ Conventional morphological networks are intersubject covariance networks constructed using a certain morphological indicator of a group of subjects. In contrast, individual-level morphological networks, such as iRSSN in our study, provide novel perspectives for individual brains and can reflect the morphological information of a single subject.^41^ The theory of iRSSN is to construct an individual structural network using radiomics, which considers all the features of texture information on structural brain MRI.^13,42^

Our data suggested that iRSSN in our healthy controls and RE patients exhibited a higher Cp but similar Lp compared with corresponding random networks across a wide range of network densities, suggestive of a small-world topology without differences between controls and RE. However, if deviations away from a small-world organization occur, that is, topological profiles either network regularization (increased Cp and Lp) or randomization (decreased Cp and Lp). In previous EEG/intracranial EEG studies, network regularization at seizure onset has been reported, with a configuration shifting towards a globally integrated process as the seizure spreads, eventually reaching a random configuration at seizure termination.^43^ Understanding such structural reorganization offers comprehensive knowledge of the neural substrates and pathophysiological mechanisms of RE.

EPC preserved an overall small-world configuration with increased Cp and decreased Lp over a wide sparsity range. In contrast with the NEPC group, iRSSN configurations in the EPC group showed a tendency towards a more regularized, ‘lattice-like’, architecture with higher Cp and high Lp. Studies of structural connectomes in temporal lobe epilepsy found that more regularized networks are spatially compact, which may facilitate recurrent excitatory activity and high-frequency oscillation.^44^ A higher Cp property in the EPC group indicated a high local efficiency in epileptic activity accumulation in the EPC hub regions, such as the nodes of the bilateral central executive network. In addition, the disease duration of the EPC group was negatively correlated with Eg, σ, and γ but positively correlated with λ and Lp. Long-term pathological processes in EPC destroy the functional orderliness of complex networks. Therefore, the function of the morphological network in terms of segregation is attenuated with increasing RE duration. In the current results, age at onset didn’t show a remarkable correlation with graph metrics, while age at diagnosis showed a negative correlation with σ and γ, implying EPC pathological network was associated with disease duration rather than age at onset. Because no other literature studies reported the graph theory in RE patients, more related evidences and results were needed to explain.

Despite showing no differences in small-worldness in the NEPC group compared to controls, a lower Lp and Cp compared to those of the EPC group indicated a shift towards a more randomized subnetwork configuration. Overall, an increasingly random structural network organization denotes reduced local efficiency but increased global efficiency.^45^ These global topological findings in the NEPC group were complemented by region-specific mapping of graph-theoretical parameters, such as hub regions with the highest Bc in the putamen for striatal-thalamo-cortical inhibition. Clustering reduction implies a weakening of focal specialization, but a reduction in path length, that is, an increase in global network efficiency, may indicate a breakdown of the balance between integration and segregation in the NEPC group. Such imbalance might explain, at least in part, the ability of epileptiform discharges to rapidly spread and be hard to reach the seizure-triggering threshold in NEPC patients. Thus, the tendency of randomness alteration in NEPC patients could facilitate the spread of epileptic activity, thereby obstructing focal abnormal synchronization.

### Limitations

First, our study lacked RE longitudinal data on the prediagnosis or prodromal phase to produce the prediction model of the occurrence of EPC in the acute phase. Early diagnosis is difficult due to the low morbidity and heterogeneity of RE patients. Second, we did not calculate other morphological features, such as cortical thickness and gyrus curvature. We want to obtain results that are close to clinical practices and MRI diagnoses in RE patients. However, future studies will involve other feature vectors to achieve more comprehensive morphological findings. Third, although the individual-level morphological network provides a way to measure interregional similarities in the alteration of RE hemisphere atrophy, in-depth studies with larger sample sizes are still needed to further reveal the role and biological meanings of morphological networks. Last, we did not assess diffusion-based structural networks and functional brain networks, which will be extremely useful for exploring individual RE differences in network dynamics and their relationship with clinical measures. However, as one of the top centres in the world for diagnosing and treating people with RE, our future work will focus on the comprehensive collection of longitudinal data in the early course of RE to provide a connectome-based early diagnosis criterion.

## Conclusion

In summary, we provide the first report to date that EPC generation in RE patients is associated with regularized connectome disruptions and not restricted to motor cortical atrophy. In addition, putamen atrophy was the most severe on the affected side, possibly resulting in diminished inhibitory effects of striatal-thalamo-cortical circuits in the EPC group. This observation makes EPC symptoms directly relevant to the disorganized structural connectome in a sample with clinically defined RE. These findings advance our understanding of the effects of EPC on large-scale brain networks and could be used to develop putamen-target neuromodulation for RE patients with EPC.

## Supplementary Material

fcae316_Supplementary_Data

## Data Availability

The data are available from the corresponding authors upon reasonable request.
