# Supplementary material for: Volume-based structural connectome of epilepsy partialis continua in Rasmussen’s encephalitis
Source: Brain Commun. 2024 Sep 20;6(5):fcae316. doi: 10.1093/braincomms/fcae316 (PMC11443448; doi:10.1093/braincomms/fcae316)
Supplement: fcae316_Supplementary_Data [file fcae316_supplementary_data.docx]

**Volumed-based Structural Connectome of Epilepsy Partialis Continua in Rasmussen’s Encephalitis**

**Table of Supplementary Contents**

**Supplemental Methods**

**Supplemental Tables**

**Table 1.** Diagnostic criteria for RE in the Part A

**Table 2.** Clinical characteristics of patients in RE group.

**Table 3.** Demographic information of healthy controls

**Table 4.** Parcellated brain regions and labels abbreviation based on Automated Anatomical Labeling (AAL) Atlas.

**Table 5.** Raw volume in the 90 regions of grey matter across groups.

**Table 6.** Group differences in GMV

**Table 7.** Hub distribution in the EPC group and the NEPC group.

**Table 8.** Modularity in the EPC group and the NEPC group.

**Table 9.** Pearson correlations between network properties and clinical information.

**Supplementary references**

**Supplemental Methods**

**Structure network construction**

**SCN Construction**

The edges corresponded to group-wise correlations between the normalized volume of each brain region. The normalized gray matter images of each data group were submitted to graph theoretical analysis. For each group, the structural correlation network was constructed by analyzing a total of 90 cortical and subcortical regions of interest (ROIs) in the Automated Anatomical Labeling template^1^ to form a 90 × 90 association matrix consisting of Pearson correlation coefficients between cortical gray matter volumes (i.e., anatomical covariance). Binary association matrices were used given the methodological concerns when comparing weighted matrices. Graph G had a network degree of E equal to the number of edges, and a network density (cost) of D = E/ [Nx (− 1)]/2 representing the ratio of existing edges relative to all possible edges. In this study, thresholds set across a range of network densities (Dmin–Dmax, Dmin = 0.3 and Dmax = 0.5 at intervals of 0.02), where means the lowest density that can permit a fully connected.

**iRSSN Construction**

Radiomics features were computed for 90 GM regions of the AAL atlas^2^ in the brain. Specifically, Radiomics has tens to thousands of feature sets, but many of these feature sets are correlated with each other. In our study, 43 representative high-order Radiomics features were selected, including Gray-level co-occurrence matrix (GLCM), Gray-level run-length matrix (GLRLM), Gray-level size zone matrix (GLSZM), Neighborhood gray-tone difference matrix (NGTDM). Vallières et al. gave the detail definition and description about above features ^3^. Each feature was then normalized by a demeaning manner (divided by the mean across different brain regions) to lessen the data range difference, and the Pearson correlation coefficients were computed between each pairwise ROIs, using the open-source code from Liu and his colleagues^4^ build individual 90×90 iRSSN matrix for every subject. In iRSSN study, thresholds of network densities were calculated and the range between 0.20 to 0.46 at intervals of 0.02.

**Topological Properties**

Measures of integration, segregation and resilience were derived from each group-specific graph whose edges were constructed on the basis of group-level correlation among the individual brain regions (nodes). A range of network thresholds based on connection density (i.e., 0.30 – 0.5 with steps of interval 0.02) was applied to generate binary undirected adjacency matrices. Computed topological measures were normalized to equivalent values derived from 20 random (‘null’) networks with the same degree distribution.

Small-worldness(σ), an indicator of ensemble topology, which is calculated as [C/Crand]/[L/Lrand], where Crand and Lrand, are the normalized clustering coefficient (γ) and the normalized characteristic path length (*λ*) of 1000 random networks, respectively. Small-world networks display greater clustering but similar path length relative to random networks. This organization represents an optimal balance between network segregation and integration^5^. Most biological networks, including neuroimaging based connectomes show σ>1, indicating efficient information transfer at a relatively low wiring cost^6^. Modularity analysis identifies subdivisions (modules) in a network that have maximal within-module connections and minimal between-module links. The algorithm was used permutation testing with 1000 permutations and the modular structure with the highest maximized modularity value was used as the representative modular structure for each group. Betweenness centrality (Bc) is the fraction of all shortest paths in the network that pass-through a given node. Network hubs are the most globally interconnected regions in a network and were defined as a region whose nodal BC was two standard deviations higher than the mean network betweenness^7^.

**Supplementary Table 1.** **Diagnostic criteria for RE in the Part A**

| **1. Clinical** | Focal seizures (with or without epilepsia partialis continua) and unilateral cortical deficit(s) |
| --- | --- |
| **2. EEG** | Unihemispheric slowing with or without epileptiform activity and unilateral seizure onset |
| **3. MRI** | Unihemispheric focal cortical atrophy and at least one of the following:   1. Gray or white matter T2/FLAIR hyperintense signal 2. Hyperintense signal or atrophy of the ipsilateral caudate head |

**Supplementary Table 2. Clinical characteristics of patients in RE group.**

| **ID** | **EPC** | **Side of affected** | **Surgery** | **Age at diagnosis**  **(years)** | **Age at onset**  **(years)** | **Sex** | **Duration**  **of RE**  **(months)** | **TIV**  **(ml)** |
| --- | --- | --- | --- | --- | --- | --- | --- | --- |
| sub01 | No EPC history, seizures many times per day(limbs) | R | 1 | 7.00 | 5.00 | F | 24.00 | 1394.99 |
| sub02 | Yes (limbs) | R | 1 | 4.00 | 3.00 | F | 10.00 | 1433.83 |
| sub03 | Yes (limbs) | R | 1 | 8.00 | 7.00 | F | 12.00 | 1466.77 |
| sub04 | Yes (limbs) | R | 1 | 4.00 | 3.00 | F | 5.00 | 1256.83 |
| sub05 | No EPC history and seizures 2-3/days(limbs) | R | 1 | 7.00 | 5.00 | F | 24.00 | 1311.46 |
| sub06 | No EPC history, seizures several times per day (limbs and month) | R | 1 | 6.00 | 5.00 | M | 4.00 | 1494.50 |
| sub07 | No EPC history, seizures 2-3/week(limbs) | R | 1 | 4.00 | 3.00 | F | 12.00 | 1386.12 |
| sub08 | No EPC history. 1-4/week (limbs) | R | 3 | 17.0 | 5.00 | F | 144.00 | 1221.01 |
| sub09 | Yes (upper limbs) | L | 1 | 4.00 | 3.00 | F | 8.00 | 1046.23 |
| sub10 | Yes (limbs and facial) | L | 1 | 8.00 | 7.00 | F | 10.00 | 1194.85 |
| sub11 | Yes (facial) | L | 1 | 13.00 | 8.00 | F | 60.00 | 1487.53 |
| sub12 | Yes (facial) | L | 1 | 17.00 | 7.00 | F | 120.00 | 1404.25 |
| sub13 | No EPC history, 2-3/week(limbs) | L | 1 | 11.00 | 8.00 | F | 36.00 | 1476.40 |
| sub14 | Yes (lower limbs) | L | 1 | 5.00 | 3.00 | M | 16.00 | 1299.88 |
| sub15 | Yes (facial) | L | 1 | 8.00 | 6.00 | M | 24.00 | 1524.22 |
| sub16 | No EPC history, 1/week(limbs) | L | 3 | 6.00 | 4.00 | M | 18.00 | 1296.94 |
| sub17 | No EPC history, 10/days(limbs) | L | 2 | 5.00 | 4.00 | F | 8.00 | 1347.05 |
| sub18 | No EPC history, several times per day(limbs) | L | 1 | 11.0 | 6.00 | M | 60.00 | 1251.31 |
| sub19 | Yes (limbs) | L | 1 | 6.00 | 4.00 | M | 24.00 | 1331.03 |
| sub20 | Yes (limbs) | R | 1 | 3.00 | 3.00 | F | 6.00 | 1257.20 |

RE Surgery 1: Hemispherectomy; 2: Multiple lobectomy; 3: No surgery. TIV = total intracranial volume; R = right; L = left; F = female; M = male.

**Supplementary Table 3.** **Demographic information of healthy controls.**

| **ID** | **Age (year)** | **Sex** | **TIV (ml)** |
| --- | --- | --- | --- |
| **Sub01** | **11** | **M** | **1439.64** |
| **Sub02** | **17** | **M** | **1663.11** |
| **Sub03** | **10** | **F** | **1384.68** |
| **Sub04** | **17** | **M** | **1768.78** |
| **Sub05** | **14** | **M** | **1556.67** |
| **Sub06** | **6** | **M** | **1323.66** |
| **Sub07** | **4** | **F** | **1294.26** |
| **Sub08** | **5** | **M** | **1322.42** |
| **Sub09** | **9** | **F** | **1322** |
| **Sub10** | **13** | **M** | **1429.71** |
| **Sub11** | **10** | **F** | **1484.52** |
| **Sub12** | **9** | **F** | **1444.51** |
| **Sub13** | **16** | **M** | **1385.1** |
| **Sub14** | **11** | **M** | **1431.44** |
| **Sub15** | **9** | **F** | **1111.51** |
| **Sub16** | **8** | **F** | **1433.31** |
| **Sub17** | **7** | **F** | **1313.51** |
| **Sub18** | **6** | **F** | **1234.4** |
| **Sub19** | **8** | **F** | **1496.83** |
| **Sub20** | **17** | **M** | **1596.36** |

TIV = total intracranial volume; F = female; M = male.

**Supplementary Table 4. The detailed brain region names of the AAL atlas (https://www.gin.cnrs.fr/en/tools/aal/).**

| **Label** | **Description** | **Label** | **Description** |
| --- | --- | --- | --- |
| AMYG-L | Amygdala_L | AMYG-R | Amygdala_R |
| ANG-L | Angular_L | ANG-R | Angular_R |
| CALC-L | Calcarine_L | CALC-R | Calcarine_R |
| CN-L | Caudate_L | CN-R | Caudate_R |
| ACC-L | Cingulum_Ant_L | ACC-R | Cingulum_Ant_R |
| MCC-L | Cingulum_Mid_L | MCC-R | Cingulum_Mid_R |
| PCC-L | Cingulum_Post_L | PCC-R | Cingulum_Post_R |
| CUN-L | Cuneus_L | CUN-R | Cuneus_R |
| IFOp-L | Frontal_Inf_Oper_L | IFOp-R | Frontal_Inf_Oper_R |
| IFOr-L | Frontal_Inf_Orb_L | IFOr-R | Frontal_Inf_Orb_R |
| IFTr-L | Frontal_Inf_Tri_L | IFTr-R | Frontal_Inf_Tri_R |
| MedFOr-L | Frontal_Med_Orb_L | MedFOr-R | Frontal_Med_Orb_R |
| MFG-L | Frontal_Mid_Orb_L | MFG-R | Frontal_Mid_Orb_R |
| MFOr-L | Frontal_Mid_L | MFOr-R | Frontal_Mid_R |
| SFG-L | Frontal_Sup_Medial_L | SFG-R | Frontal_Sup_Medial_R |
| MedSF-L | Frontal_Sup_Orb_L | MedSF-R | Frontal_Sup_Orb_R |
| SFOr-L | Frontal_Sup_L | SFOr-R | Frontal_Sup_R |
| FG-L | Fusiform_L | FG-R | Fusiform_R |
| HSHL-L | Heschel_L | HSHL-R | Heschel_R |
| HIPP-L | Hippocampus_L | HIPP-R | Hippocampus_R |
| INS-L | Insula_L | INS-R | Insula_R |
| LNG-L | Lingual_L | LNG-R | Lingual_R |
| IOG-L | Occipital_Inf_L | IOG-R | Occipital_Inf_R |
| MOG-L | Occipital_Mid_L | MOG-R | Occipital_Mid_R |
| SOG-L | Occipital_Sup_L | SOG-R | Occipital_Sup_R |
| OFB-L | Olfactory_L | OFB-R | Olfactory_R |
| PLD-L | Pallidum_L | PLD-R | Pallidum_R |
| PCL-L | Paracentral_Lobule_L | PCL-R | Paracentral_Lobule_R |
| PHIP-L | Parahippocampal_L | PHIP-R | Parahippocampal_R |
| IPL-L | Parietal_Inf_L | IPL-R | Parietal_Inf_R |
| SPL-L | Parietal_Sup_L | SPL-R | Parietal_Sup_R |
| PoCG-L | Postcentral_L | PoCG-R | Postcentral_R |
| PrCG-L | Precentral_L | PrCG-R | Precentral_R |
| PCUN-L | Precuneus_L | PCUN-R | Precuneus_R |
| PUT-L | Putamen_L | PUT-R | Putamen_R |
| REC-L | Rectus_L | REC-R | Rectus_R |
| RLN-L | Rolandic_Oper_L | RLN-R | Rolandic_Oper_R |
| SMA-L | Supp_Motor_Area_L | SMA-R | Supp_Motor_Area_R |
| SMG-L | SupraMarginal_L | SMG-R | SupraMarginal_R |
| ITG-L | Temporal_Inf_L | ITG-R | Temporal_Inf_R |
| MTG-L | Temporal_Mid_L | MTG-R | Temporal_Mid_R |
| MTP-L | Temporal_Pole_Mid_L | MTP-R | Temporal_Pole_Mid_R |
| STP-L | Temporal_Pole_Sup_L | STP-R | Temporal_Pole_Sup_R |
| STG-L | Temporal_Sup_L | STG-R | Temporal_Sup_R |
| THL-L | Thalamus_L | THL-R | Thalamus_R |

**Supplementary Table 5. GM raw volume**

| AAL_Lables | HC | EPC | NPEC |
| --- | --- | --- | --- |
| 1. 'Amygdala_L' | 0.57±0.08 | 0.53±0.07 | 0.52±0.04 |
| 1. 'Amygdala_R' | 0.53±0.07 | 0.43±0.07 | 0.41±0.06 |
| 1. 'Angular_L' | 0.43±0.04 | 0.42±0.04 | 0.41±0.07 |
| 1. 'Angular_R' | 0.4±0.05 | 0.34±0.04 | 0.3±0.06 |
| 1. 'Calcarine_L' | 0.34±0.06 | 0.32±0.03 | 0.33±0.05 |
| 1. 'Calcarine_R' | 0.33±0.07 | 0.28±0.03 | 0.26±0.05 |
| 1. 'Caudate_L' | 0.4±0.03 | 0.36±0.03 | 0.35±0.03 |
| 1. 'Caudate_R' | 0.42±0.03 | 0.3±0.1 | 0.31±0.07 |
| 1. 'Cingulum_Ant_L' | 0.48±0.06 | 0.43±0.02 | 0.42±0.04 |
| 1. 'Cingulum_Ant_R' | 0.43±0.05 | 0.36±0.04 | 0.32±0.08 |
| 1. 'Cingulum_Mid_L' | 0.49±0.04 | 0.45±0.04 | 0.46±0.05 |
| 1. 'Cingulum_Mid_R' | 0.45±0.04 | 0.38±0.04 | 0.36±0.06 |
| 1. 'Cingulum_Post_L' | 0.41±0.05 | 0.38±0.04 | 0.4±0.05 |
| 1. 'Cingulum_Post_R' | 0.32±0.03 | 0.26±0.04 | 0.26±0.02 |
| 1. 'Cuneus_L' | 0.34±0.04 | 0.33±0.04 | 0.31±0.05 |
| 1. 'Cuneus_R' | 0.33±0.05 | 0.28±0.04 | 0.27±0.04 |
| 1. 'Frontal_Inf_Oper_L' | 0.38±0.05 | 0.37±0.05 | 0.36±0.04 |
| 1. 'Frontal_Inf_Oper_R' | 0.41±0.05 | 0.3±0.06 | 0.28±0.08 |
| 1. 'Frontal_Inf_Orb_L' | 0.4±0.05 | 0.38±0.03 | 0.39±0.04 |
| 1. 'Frontal_Inf_Orb_R' | 0.39±0.05 | 0.28±0.06 | 0.27±0.08 |
| 1. 'Frontal_Inf_Tri_L' | 0.38±0.05 | 0.35±0.04 | 0.35±0.05 |
| 1. 'Frontal_Inf_Tri_R' | 0.35±0.04 | 0.27±0.05 | 0.26±0.07 |
| 1. 'Frontal_Med_Orb_L' | 0.43±0.08 | 0.42±0.03 | 0.41±0.08 |
| 1. 'Frontal_Med_Orb_R' | 0.42±0.09 | 0.37±0.05 | 0.33±0.07 |
| 1. 'Frontal_Mid_L' | 0.38±0.07 | 0.36±0.05 | 0.36±0.05 |
| 1. 'Frontal_Mid_Orb_L' | 0.39±0.08 | 0.39±0.05 | 0.38±0.06 |
| 1. 'Frontal_Mid_Orb_R' | 0.38±0.07 | 0.32±0.07 | 0.28±0.08 |
| 1. 'Frontal_Mid_R' | 0.38±0.06 | 0.32±0.05 | 0.29±0.05 |
| 1. 'Frontal_Sup_L' | 0.32±0.06 | 0.32±0.04 | 0.31±0.04 |
| 1. 'Frontal_Sup_Medial_L' | 0.36±0.05 | 0.36±0.02 | 0.35±0.05 |
| 1. 'Frontal_Sup_Medial_R' | 0.36±0.07 | 0.33±0.04 | 0.3±0.05 |
| 1. 'Frontal_Sup_Orb_L' | 0.39±0.07 | 0.4±0.05 | 0.39±0.05 |
| 1. 'Frontal_Sup_Orb_R' | 0.39±0.06 | 0.33±0.06 | 0.29±0.08 |
| 1. 'Frontal_Sup_R' | 0.33±0.05 | 0.29±0.05 | 0.27±0.05 |
| 1. 'Fusiform_L' | 0.48±0.04 | 0.43±0.05 | 0.46±0.05 |
| 1. 'Fusiform_R' | 0.49±0.05 | 0.38±0.05 | 0.41±0.04 |
| 1. 'Heschel_R' | 0.44±0.05 | 0.34±0.1 | 0.31±0.08 |
| 1. 'Heschl_L' | 0.44±0.05 | 0.4±0.05 | 0.43±0.05 |
| 1. 'Hippocampus_L' | 0.42±0.05 | 0.36±0.09 | 0.38±0.05 |
| 1. 'Hippocampus_R' | 0.41±0.05 | 0.27±0.07 | 0.28±0.06 |
| 1. 'Insula_L' | 0.5±0.05 | 0.47±0.03 | 0.48±0.05 |
| 1. 'Insula_R' | 0.53±0.05 | 0.39±0.07 | 0.36±0.1 |
| 1. 'Lingual_L' | 0.39±0.04 | 0.36±0.03 | 0.39±0.05 |
| 1. 'Lingual_R' | 0.37±0.04 | 0.32±0.03 | 0.32±0.05 |
| 1. 'Occipital_Inf_L' | 0.37±0.04 | 0.34±0.04 | 0.37±0.07 |
| 1. 'Occipital_Inf_R' | 0.3±0.05 | 0.3±0.05 | 0.27±0.06 |
| 1. 'Occipital_Mid_L' | 0.37±0.06 | 0.35±0.04 | 0.35±0.07 |
| 1. 'Occipital_Mid_R' | 0.36±0.05 | 0.34±0.05 | 0.29±0.05 |
| 1. 'Occipital_Sup_L' | 0.29±0.05 | 0.29±0.04 | 0.28±0.05 |
| 1. 'Occipital_Sup_R' | 0.3±0.04 | 0.28±0.04 | 0.26±0.04 |
| 1. 'Olfactory_L' | 0.55±0.06 | 0.48±0.05 | 0.49±0.05 |
| 1. 'Olfactory_R' | 0.55±0.06 | 0.43±0.07 | 0.39±0.08 |
| 1. 'Pallidum_L' | 0.33±0.04 | 0.32±0.03 | 0.3±0.04 |
| 1. 'Pallidum_R' | 0.38±0.04 | 0.26±0.08 | 0.26±0.08 |
| 1. 'Paracentral_Lobule_L' | 0.29±0.04 | 0.26±0.03 | 0.29±0.04 |
| 1. 'Paracentral_Lobule_R' | 0.31±0.03 | 0.28±0.04 | 0.27±0.08 |
| 1. 'Parahippocampal_L' | 0.4±0.04 | 0.36±0.05 | 0.37±0.03 |
| 1. 'Parahippocampal_R' | 0.47±0.05 | 0.35±0.06 | 0.36±0.04 |
| 1. 'Parietal_Inf_L' | 0.44±0.04 | 0.41±0.04 | 0.4±0.06 |
| 1. 'Parietal_Inf_R' | 0.42±0.05 | 0.37±0.06 | 0.34±0.05 |
| 1. 'Parietal_Sup_L' | 0.35±0.04 | 0.34±0.04 | 0.33±0.05 |
| 1. 'Parietal_Sup_R' | 0.28±0.04 | 0.27±0.03 | 0.25±0.03 |
| 1. 'Postcentral_L' | 0.34±0.03 | 0.32±0.03 | 0.33±0.05 |
| 1. 'Postcentral_R' | 0.33±0.03 | 0.29±0.03 | 0.28±0.06 |
| 1. 'Precentral_L' | 0.35±0.04 | 0.32±0.02 | 0.33±0.02 |
| 1. 'Precentral_R' | 0.33±0.03 | 0.27±0.05 | 0.25±0.06 |
| 1. 'Precuneus_L' | 0.4±0.03 | 0.37±0.03 | 0.38±0.05 |
| 1. 'Precuneus_R' | 0.41±0.04 | 0.34±0.05 | 0.33±0.05 |
| 1. 'Putamen_L' | 0.55±0.05 | 0.52±0.03 | 0.51±0.06 |
| 1. 'Putamen_R' | 0.55±0.05 | 0.39±0.09 | 0.39±0.09 |
| 1. 'Rectus_L' | 0.45±0.05 | 0.41±0.04 | 0.42±0.04 |
| 1. 'Rectus_R' | 0.46±0.05 | 0.38±0.05 | 0.33±0.08 |
| 1. 'Rolandic_Oper_L' | 0.43±0.04 | 0.41±0.05 | 0.43±0.05 |
| 1. 'Rolandic_Oper_R' | 0.44±0.04 | 0.33±0.07 | 0.32±0.11 |
| 1. 'Supp_Motor_Area_L' | 0.38±0.04 | 0.34±0.03 | 0.35±0.04 |
| 1. 'Supp_Motor_Area_R' | 0.36±0.03 | 0.31±0.04 | 0.3±0.09 |
| 1. 'SupraMarginal_L' | 0.42±0.05 | 0.4±0.06 | 0.4±0.07 |
| 1. 'SupraMarginal_R' | 0.41±0.03 | 0.36±0.05 | 0.33±0.07 |
| 1. 'Temporal_Inf_L' | 0.45±0.05 | 0.39±0.05 | 0.4±0.04 |
| 1. 'Temporal_Inf_R' | 0.42±0.04 | 0.34±0.06 | 0.34±0.04 |
| 1. 'Temporal_Mid_L' | 0.45±0.05 | 0.42±0.04 | 0.43±0.05 |
| 1. 'Temporal_Mid_R' | 0.44±0.04 | 0.35±0.06 | 0.34±0.06 |
| 1. 'Temporal_Pole_Mid_L' | 0.39±0.07 | 0.37±0.06 | 0.36±0.03 |
| 1. 'Temporal_Pole_Mid_R' | 0.37±0.04 | 0.26±0.06 | 0.27±0.04 |
| 1. 'Temporal_Pole_Sup_L' | 0.37±0.05 | 0.33±0.04 | 0.34±0.02 |
| 1. 'Temporal_Pole_Sup_R' | 0.37±0.04 | 0.26±0.06 | 0.26±0.04 |
| 1. 'Temporal_Sup_L' | 0.42±0.04 | 0.4±0.03 | 0.41±0.05 |
| 1. 'Temporal_Sup_R' | 0.41±0.03 | 0.31±0.06 | 0.29±0.05 |
| 1. 'Thalamus_L' | 0.42±0.06 | 0.36±0.03 | 0.4±0.05 |
| 1. 'Thalamus_R' | 0.44±0.05 | 0.34±0.05 | 0.33±0.1 |

**Supplementary Table** **6 Group differences in GM volume**

| **Region**  **(AAL atlas)** | **Side** | **Coordinate (MNI)** | | | **Cluster size** | **Maximal *t* value** |
| --- | --- | --- | --- | --- | --- | --- |
|  |  | **X** | **Y** | **Z** |  |  |
| **EPC<Controls** | | | | | | |
| Putamen | R | 27 | 18 | 0 | 2024 | -9.18 |
| Caudate* | R | 6 | 23 | 0 |  | -7.41 |
| Pallidum* | R | 18 | 9 | 2 |  | -6.11 |
| Thalamus* | R | 3 | -9 | 11 |  | -7.37 |
| Calcarine | R | 15 | -84 | 13.5 | 17 | -4.13 |
| Precuneus | R | 22.5 | -69 | 31.5 | 583 | -5.96 |
| Cingulum_Mid | R | 3 | -28.5 | 43.5 | 1242 | -6.14 |
| Cingulum_Ant | R | 4 | 32 | 29 | 271 | -4.71 |
| Precentral | R | 28.5 | 0 | 45 | 176 | -6.99 |
| Frontal_Mid | R | 39 | 19.5 | 51 | 121 | -4.50 |
| **NEPC<Controls** | | | | | | |
| Temporal_Mid | R | 51 | -22.5 | -16.5 | 4134 | -9.88 |
| Putamen* | R | 28 | 19 | 6 |  | -7.12 |
| Caudate* | R | 15 | 12 | 17 |  | -5.71 |
| Pallidum* | R | 20 | 7 | 3 |  | -6.14 |
| Thalamus* | R | 5 | -6 | 8 |  | -5.11 |
| Frontal_Sup | R | 15 | 18 | 51 | 145 | -5.1389 |
| Precuneus | R | 10.5 | -57 | 33 | 562 | -5.2575 |
| Cuneus | R | 12 | -81 | 22.5 | 50 | -4.6146 |
| Cingulum_Mid | R | 12 | -31.5 | 34.5 | 1239 | -6.2506 |
| Frontal_Mid | R | 31 | 0 | 53 | 299 | -9.0447 |
| Precentral | R | 36 | -21 | 51 | 294 | -6.3231 |

VBM results of the group-factor ANOVA comparison (controlled for age, sex, TIV, voxel threshold p<0.001, cluster threshold p<0.05, GFR correction). There was no cluster survived in the comparison between EPC and NEPC groups. *: a local maximum within the above cluster; R = right.

**Supplementary Table 7. Hub regions of high betweenness centrality relative to random networks**

| **Hub（AAL）** | **Normalized betweenness centrality** | **Modularity** | **Functional classification** | **Anatomical**  **classification** |
| --- | --- | --- | --- | --- |
| **EPC** |  |  |  |  |
| Cingulum_Mid_L | 9.10 | 4 | Paralimbic | Frontal |
| Frontal_Inf_Orb_L | 7.14 | 1 | Paralimbic | Prefrontal |
| Frontal_Sup_R | 6.62 | 2 | Association | Prefrontal |
| Parietal_Sup_L | 6.05 | 4 | Association | Parietal |
| Parietal_Sup_R | 7.00 | 1 | Association | Parietal |
| **NEPC** |  |  |  |  |
| Angular_R | 8.99 | 3 | Association | Parietal |
| Cingulum_Ant_R | 9.02 | 1 | Paralimbic | Prefrontal |
| Fusiform_R | 7.88 | 3 | Association | Temporal |
| Precentral_R | 7.92 | 1 | Primary | Frontal |
| Putamen_L | 9.10 | 3 | Subcortical | Subcortical |
| Putamen_R  Temporal_Mid_R | 7.25  7.86 | 1  3 | Subcortical  Association | Subcortical  Temporal |

Hubs are defined as those with a nodal betweenness centrality that was 2 SD above the mean nodal betweenness centrality of all cortical regions.

**Supplementary Table 8. Modularity in the EPC group and the NEPC group.**

| **EPC Modularity** | | | |
| --- | --- | --- | --- |
| **Mondule1** | **Mondule2** | **Mondule3** | **Mondule4** |
| 'Caudate_L' | 'Calcarine_L' | 'Amygdala_R' | 'Amygdala_L' |
| 'Caudate_R' | 'Calcarine_R' | 'Angular_R' | 'Angular_L' |
| 'Cingulum_Ant_R' | 'Cingulum_Mid_R' | 'Cingulum_Ant_L' | 'Cingulum_Mid_L' |
| 'Frontal_Inf_Oper_R' | 'Cuneus_L' | 'Cingulum_Post_R' | 'Cingulum_Post_L' |
| 'Frontal_Inf_Orb_L' | 'Cuneus_R' | 'Frontal_Med_Orb_R' | 'Fusiform_L' |
| 'Frontal_Inf_Orb_R' | 'Frontal_Inf_Oper_L' | 'Frontal_Sup_Medial_R' | 'Hippocampus_L' |
| 'Frontal_Inf_Tri_R' | 'Frontal_Inf_Tri_L' | 'Fusiform_R' | 'Parahippocampal_L' |
| 'Frontal_Mid_L' | 'Frontal_Med_Orb_L' | 'Heschel_R' | 'Parietal_Sup_L' |
| 'Frontal_Mid_Orb_L' | 'Frontal_Sup_Medial_L' | 'Heschl_L' | 'Temporal_Inf_L' |
| 'Frontal_Mid_Orb_R' | 'Lingual_L' | 'Hippocampus_R' | 'Temporal_Mid_L' |
| 'Frontal_Mid_R' | 'Lingual_R' | 'Insula_L' | 'Temporal_Pole_Mid_L' |
| 'Frontal_Sup_L' | 'Occipital_Inf_L' | 'Insula_R' | 'Temporal_Pole_Sup_L' |
| 'Frontal_Sup_Orb_L' | 'Occipital_Inf_R' | 'Parahippocampal_R' |  |
| 'Frontal_Sup_Orb_R' | 'Occipital_Mid_L' | 'Parietal_Inf_R' |  |
| 'Frontal_Sup_R' | 'Occipital_Sup_L' | 'Putamen_L' |  |
| 'Occipital_Mid_R' | 'Occipital_Sup_R' | 'Rolandic_Oper_L' |  |
| 'Olfactory_L' | 'Paracentral_Lobule_R' | 'Rolandic_Oper_R' |  |
| 'Olfactory_R' | 'Parietal_Inf_L' | 'SupraMarginal_R' |  |
| 'Pallidum_L' | 'Postcentral_L' | 'Temporal_Inf_R' |  |
| 'Pallidum_R' | 'Postcentral_R' | 'Temporal_Mid_R' |  |
| 'Paracentral_Lobule_L' | 'Precuneus_L' | 'Temporal_Pole_Mid_R' |  |
| 'Parietal_Sup_R' | 'Precuneus_R' | 'Temporal_Pole_Sup_R' |  |
| 'Precentral_L' | 'Supp_Motor_Area_L' | 'Temporal_Sup_R' |  |
| 'Precentral_R' |  |  |  |
| 'Putamen_R' |  |  |  |
| 'Rectus_L' |  |  |  |
| 'Rectus_R' |  |  |  |
| 'Supp_Motor_Area_R' |  |  |  |
| 'SupraMarginal_L' |  |  |  |
| 'Temporal_Sup_L' |  |  |  |
| 'Thalamus_L' |  |  |  |
| 'Thalamus_R' |  |  |  |

| **NEPC Modularity** | | | |
| --- | --- | --- | --- |
| **Mondule1** | **Mondule2** | **Mondule3** | **Mondule4** |
| 'Caudate_R' | 'Amygdala_L' | Amygdala_R' | 'Cingulum_Post_R' |
| 'Cingulum_Ant_R' | 'Angular_L' | 'Angular_R' | 'Paracentral_Lobule_L' |
| 'Cingulum_Mid_R' | 'Calcarine_L' | 'Calcarine_R' | 'Temporal_Pole_Mid_L' |
| 'Frontal_Inf_Oper_R' | 'Cingulum_Ant_L' | 'Caudate_L' |  |
| 'Frontal_Inf_Orb_R' | 'Cingulum_Mid_L' | 'Cuneus_R' |  |
| 'Frontal_Inf_Tri_R' | 'Cingulum_Post_L' | 'Fusiform_R' |  |
| 'Frontal_Med_Orb_R' | 'Cuneus_L' | 'Heschel_R' |  |
| 'Frontal_Mid_Orb_R' | 'Frontal_Inf_Oper_L' | 'Lingual_R' |  |
| 'Frontal_Mid_R' | 'Frontal_Inf_Orb_L' | 'Occipital_Inf_R' |  |
| 'Frontal_Sup_Medial_R' | 'Frontal_Inf_Tri_L' | 'Occipital_Mid_R' |  |
| 'Frontal_Sup_Orb_R' | 'Frontal_Med_Orb_L' | 'Occipital_Sup_R' |  |
| 'Frontal_Sup_R' | 'Frontal_Mid_L' | 'Olfactory_L' |  |
| 'Hippocampus_R' | 'Frontal_Mid_Orb_L' | 'Pallidum_L' |  |
| 'Insula_R' | 'Frontal_Sup_L' | 'Parahippocampal_R' |  |
| 'Olfactory_R' | 'Frontal_Sup_Medial_L' | 'Parietal_Inf_R' |  |
| 'Pallidum_R' | 'Frontal_Sup_Orb_L' | 'Parietal_Sup_R' |  |
| 'Paracentral_Lobule_R' | 'Fusiform_L' | 'Precuneus_R' |  |
| 'Postcentral_R' | 'Heschl_L' | 'Putamen_L' |  |
| 'Precentral_R' | 'Hippocampus_L' | 'SupraMarginal_R' |  |
| 'Putamen_R' | 'Insula_L' | 'Temporal_Inf_R' |  |
| 'Rectus_R' | 'Lingual_L' | 'Temporal_Mid_R' |  |
| 'Rolandic_Oper_R' | 'Occipital_Inf_L' | 'Temporal_Pole_Mid_R' |  |
| 'Supp_Motor_Area_R' | 'Occipital_Mid_L' | 'Temporal_Pole_Sup_R' |  |
|  | 'Occipital_Sup_L' | 'Temporal_Sup_R' |  |
|  | 'Parahippocampal_L' | 'Thalamus_L' |  |
|  | 'Parietal_Inf_L' | 'Thalamus_R' |  |
|  | 'Parietal_Sup_L' |  |  |
|  | 'Postcentral_L' |  |  |
|  | 'Precentral_L' |  |  |
|  | 'Precuneus_L' |  |  |
|  | 'Rectus_L' |  |  |
|  | 'Rolandic_Oper_L' |  |  |
|  | 'Supp_Motor_Area_L' |  |  |
|  | 'SupraMarginal_L' |  |  |
|  | 'Temporal_Inf_L' |  |  |
|  | 'Temporal_Mid_L' |  |  |
|  | 'Temporal_Pole_Sup_L' |  |  |
|  | 'Temporal_Sup_L' |  |  |

**Supplementary Table 9. Association between graph metrics and clinical information in RE patient groups**

| **EPC group** | **Correlation coefficient** | **σ** | **λ** | **γ** | **Lp** | **Cp** | **Eg** |
| --- | --- | --- | --- | --- | --- | --- | --- |
| **Disease course** | *r* | -0.783 | 0.683 | -0.775 | 0.714 | 0.039 | -0.708 |
|  | *P* | 0.004 | 0.021 | 0.005 | 0.014 | 0.910 | 0.015 |
| **Age at diagnosis** | *r* | -0.674 | 0.526 | -0.674 | 0.564 | -0.111 | -0.556 |
|  | *P* | 0.023 | 0.096 | 0.023 | 0.071 | 0.747 | 0.076 |
| **Age at onset** | *r* | -0.2635 | 0.1974 | -0.3332 | 0.1620 | -0.3241 | -0.1882 |
|  | *P* | 0.4337 | 0.5606 | 0.3167 | 0.6342 | 0.3308 | 0.5795 |

| **NEPC group** | **Correlation coefficient** | **σ** | **λ** | **γ** | **Lp** | **Cp** | **Eg** |
| --- | --- | --- | --- | --- | --- | --- | --- |
| **Disease course** | *r* | -0.043 | 0.103 | -0.052 | -0.044 | -0.017 | 0.193 |
|  | *P* | 0.913 | 0.792 | 0.895 | 0.910 | 0.967 | 0.619 |
| **Age at diagnosis** | *r* | -0.070 | 0.036 | -0.071 | 0.013 | 0.060 | 0.134 |
|  | *P* | 0.857 | 0.926 | 0.857 | 0.975 | 0.879 | 0.731 |
| **Age at onset** | *r* | 0.106 | -0.0947 | -0.1734 | -0.1687 | -0.1497 | 0.1279 |
|  | *P* | 0.7858 | 0.8085 | 0.6555 | 0.6644 | 0.7006 | 0.7430 |

*r*, Pearson correlation coefficient. γ = normalized clustering coefficient; λ = normalized path length; σ = small-world index; Lp = characteristic path length; Cp = clustering coefficient; Eg = global efficient.

**Supplementary references**

1. Tzourio-Mazoyer N, Landeau B, Papathanassiou D, et al. Automated Anatomical Labeling of Activations in SPM Using a Macroscopic Anatomical Parcellation of the MNI MRI Single-Subject Brain. *NeuroImage*. 2002/01/01/ 2002;15(1):273-289. doi:<https://doi.org/10.1006/nimg.2001.0978>

2. Tzourio-Mazoyer N, Landeau B, Papathanassiou D, et al. Automated anatomical labeling of activations in SPM using a macroscopic anatomical parcellation of the MNI MRI single-subject brain. *Neuroimage*. Jan 2002;15(1):273-89. doi:10.1006/nimg.2001.0978

3. Vallières M, Freeman CR, Skamene SR, El Naqa I. A radiomics model from joint FDG-PET and MRI texture features for the prediction of lung metastases in soft-tissue sarcomas of the extremities. *Phys Med Biol*. Jul 21 2015;60(14):5471-96. doi:10.1088/0031-9155/60/14/5471

4. Liu H, Ma Z, Wei L, et al. A radiomics-based brain network in T1 images: construction, attributes, and applications. *Cereb Cortex*. Jan 31 2024;34(2)doi:10.1093/cercor/bhae016

5. Watts DJ, Strogatz SH. Collective dynamics of 'small-world' networks. *Nature*. Jun 4 1998;393(6684):440-2. doi:10.1038/30918

6. Humphries MD, Gurney K. Network 'small-world-ness': a quantitative method for determining canonical network equivalence. *PLoS One*. Apr 30 2008;3(4):e0002051. doi:10.1371/journal.pone.0002051

7. Hosseini SM, Hoeft F, Kesler SR. GAT: a graph-theoretical analysis toolbox for analyzing between-group differences in large-scale structural and functional brain networks. *PLoS One*. 2012;7(7):e40709. doi:10.1371/journal.pone.0040709
